# New β-Lactam/β-Lactamase Inhibitor Combination Antibiotics

**DOI:** 10.3390/pathogens14040307

**Published:** 2025-03-24

**Authors:** Maria Sargianou, Panagiotis Stathopoulos, Christos Vrysis, Iva D. Tzvetanova, Matthew E. Falagas

**Affiliations:** 1 Alfa Institute of Biomedical Sciences, 151 23 Athens, Greece; m.sargianou@aibs.gr (M.S.); p.stathopoulos@aibs.gr (P.S.); c.vrysis@aibs.gr (C.V.); 2 Hygeia Hospital, 151 23 Athens, Greece; 3 School of Medicine, European University Cyprus, 2404 Nicosia, Cyprus; i.tzvetanova@euc.ac.cy; 4 Department of Medicine, Tufts University School of Medicine, Boston, MA 02111, USA

**Keywords:** antimicrobial resistance, aztreonam/avibactam, β-lactam/β-lactamase inhibitor combination agents, carbapenem-resistant *Acinetobacter baumannii* (CRAB), cefepime/enmetazobactam, extended-spectrum β-lactamases (ESBLs), sulbactam/durlobactam, metallo-β-lactamases (MBLs), carbapenem-resistant Enterobacterales

## Abstract

The growing problem of infections due to pathogens with antimicrobial resistance, especially Gram-negative bacteria, has led to the development of new β-lactam/β-lactamase inhibitor combination antibiotics. During the last 2 years from the writing of this article, cefepime/enmetazobactam, aztreonam/avibactam, and sulbactam/durlobactam were approved for use in clinical practice. Cefepime/enmetazobactam targets extended-spectrum β-lactamase (ESBL)-producing *Pseudomonas aeruginosa* and Enterobacterales. It is indicated for the treatment of patients with complicated urinary tract infections, including pyelonephritis, in Europe and the USA, and also for hospital-acquired pneumonia, ventilator-associated pneumonia, and bacteremia associated with those infections (only in Europe). The antimicrobial spectrum of aztreonam/avibactam includes carbapenem-resistant Enterobacterales. Aztreonam/avibactam is indicated for the treatment of adult patients who suffer from complicated intra-abdominal infections, complicated urinary tract infections including pyelonephritis, hospital-acquired pneumonia, and ventilator-associated pneumonia due to aerobic Gram-negative infections with limited therapeutic options. Sulbactam/durlobactam, a combination of 2 β-lactamase inhibitors, is indicated for the treatment of adult patients with hospital-acquired bacterial pneumonia and ventilator-associated bacterial pneumonia due to the *Acinetobacter baumannii–calcoaceticus* complex [including carbapenem-resistant *Acinetobacter baumannii* (CRAB) infections].

## 1. Introduction

β-lactam antibiotics are among the most commonly prescribed antimicrobials worldwide, making up 65% of the global antibiotics market with annual sales of approximately $15 billion [1]. These antibiotics share a β-lactam ring in their chemical structure and are classified into four main categories: penicillin and its derivatives, cephalosporins, carbapenems, and monobactams. Their clinical indications and antimicrobial spectra vary across subclasses, generations, or even individual molecules [2].

The primary mechanism of action involves inhibiting bacterial-cell-wall synthesis through the acetylation of transpeptidases that catalyze peptide cross-linking into the peptidoglycan lattice, which is essential for bacterial-cell-wall integrity. These transpeptidases are also known as penicillin-binding proteins (PBPs) [2]. One mechanism of resistance that has evolved is through the production of β-lactamases, which are enzymes that cleave the β-lactam ring and neutralize these antibiotics.

Several β-lactamase inhibitors have been developed to counteract the activity of β-lactamases. These inhibitors are either competitive, acting as false substrates of β-lactamases, or non-competitive, irreversibly and chemically modifying β-lactamase active sites. However, the widespread use of β-lactam/β-lactamase inhibitor combination antibiotics has exerted selective pressure, driving the evolution of β-lactamases capable of evading inhibition [3]. The increasing resistance of Gram-negative species, including *Acinetobacter baumannii*, *Pseudomonas aeruginosa*, and *Klebsiella pneumoniae*, is more evident than ever in clinical practice. Furthermore, the emergence of pathogens producing extended-spectrum β-lactamases (ESBLs), as well as ampicillinase C (AmpC) and metallo-β-lactamases (MBLs), has raised significant concerns, prompting the continuous development of newer β-lactam/β-lactamase inhibitor combination agents [4].

ESBLs are enzymes that cause resistance to many antibiotics, including most β-lactam antibiotics (penicillins, cephalosporins, and aztreonam). Patients with infections due to ESBL-producing Enterobacterales may have poor outcomes and are commonly treated with carbapenems. MBLs are enzymes that catalyze the hydrolysis of β-lactam drugs, including carbapenems. The scarcity of clinically available inhibitors of MBLs and the considerable dissemination of the MBL genes between Gram-negative bacteria have led to devastating clinical consequences of infections due to pathogens with this resistance mechanism. AmpC, a group of β-lactamases produced by some Enterobacterales and a few other bacteria, may cause resistance to most penicillins, β-lactam/β-lactamase inhibitor combinations, and other antibiotics.

## 2. New β-Lactam/β-Lactamase Inhibitor Combination Antibiotics

In this review, we focus on β-lactam/β-lactamase inhibitor combinations that have been recently introduced into clinical practice. Three β-lactam/β-lactamase inhibitor combination antimicrobial agents were approved during the last 2 years, specifically cefepime/enmetazobactam, aztreonam/avibactam, and sulbactam/durlobactam. Table 1 presents the basic characteristics of these new β-lactam/β-lactamase inhibitor combination agents, including antibiotic class, antimicrobial spectrum, and approved indications. Table 2 presents the recommended dosage regimens based on the renal function of people taking these new β-lactam/β-lactamase inhibitor combination antibiotics.

## 3. Cefepime/Enmetazobactam

Cefepime is a fourth-generation cephalosporin with a broad-spectrum coverage against Gram-positive and Gram-negative bacteria, including good antipseudomonal activity [5]. The ability of this agent to penetrate the cell wall of pathogens and to inhibit PBP-dependent peptidoglycan synthesis provides cefepime with bactericidal ability [6]. Cefepime is generally resistant to β-lactamases, including most of the AmpC [7]. Still, it can be hydrolyzed by ESBLs and carbapenem-hydrolyzing β-lactamases [6].

The spread of multidrug-resistant (MDR) bacteria, including the rising public health problem of clinical isolates with antimicrobial resistance to β-lactam antibiotics, has led to efforts to develop new β-lactam/β-lactamase inhibitor combination antimicrobial agents [8,9]. Given the extensive antimicrobial spectrum of cefepime (and the ability to withstand hydrolysis by AmpC), scientists have tried to develop new β-lactamase inhibitors that target cefepime-hydrolyzing enzymes.

Enmetazobactam, a penicillin acid sulfone, is a recently released β-lactamase inhibitor that mostly targets ESBLs [10]. First invented in 2008 by Orchid Pharma (India), enmetazobactam was later licensed out to Allecra Therapeutics for further development [11]. Like most (but not all) β-lactamase inhibitors, enmetazobactam does not have a direct antimicrobial effect [12]. Enmetazobactam is a tazobactam derivative that, unlike its parent drug, exhibits significant activity against class A β-lactamases, including ESBLs, clavulanic-acid-resistant β-lactamases, and carbapenemases [and some of the class A *Klebsiella pneumoniae* carbapenemase (KPC), but not always] [13,14]. However, the antimicrobial spectrum of enmetazobactam is more limited than that of other new β-lactamase inhibitors, e.g., relebactam and avibactam [15], since it is inactive against class B, C, and most D β-lactamases. Notably, enmetazobactam showed limited inhibitory activity for OXA-1, a class D oxacillinase [13].

Cefepime is sensitive to ESBLs, which are inhibited by enmetazobactam, making enmetazobactam the perfect combinatory agent for cefepime. Moreover, the two agents share similar pharmacokinetic profiles [13]. The new combination is currently recommended against *Escherichia coli*, *Klebsiella pneumoniae*, *Pseudomonas aeruginosa*, *Proteus mirabilis*, and *Enterobacter cloacae* infections (Table 1) [16]. Recent studies propose to use cefepime/enmetazobactam only if the MIC of the pathogen is ≤8 μg/mL [17,18]. Mechanisms of resistance have been observed, and they occur mainly via the overexpression of efflux pumps, changes in PBPs, and mutations in the outer membrane porins, which are responsible for drug entry [19].

The pharmaceutical company Allecra (Weil am Rhein, Freiburg, Germany) manufactured cefepime/enmetazobactam, which is sold under the brand name Exblifep^®^ [14]. An agreement with Advanz Pharma was signed to commercialize the medication in the European Union, the United Kingdom, Switzerland, and Norway [20]. In February 2024, cefepime/enmetazobactam was approved by the Food and Drug Administration (FDA) for adults with complicated urinary tract infections (cUTIs) including pyelonephritis, while in Europe it has also been approved for adults with hospital-acquired pneumonia (HAP), ventilator-associated pneumonia (VAP), and bacteremia associated with those infections (March 2024) [21,22] (Table 1).

In Europe, the approval of cefepime/enmetazobactam for patients with HAP, VAP, and bacteremia associated with those conditions has been granted based on the indications for cefepime alone, studies evaluating the in vivo efficacy of cefepime/enmetazobactam in animal models with pneumonia, and on a small study enrolling 19 healthy individuals, which showed that cefepime/enmetazobactam can penetrate the lungs [16,23]. Despite the limited available clinical data on the effectiveness of cefepime/enmetazobactam in patients with HAP, VAP, and bacteremia, associated with these infections, the medication was approved in Europe for those indications (under additional monitoring), due to the unmet need for new therapeutic agents in an era of constantly rising MDR pathogens [21,23]. More clinical studies are needed to evaluate the efficacy of the novel antimicrobial combination in these clinical indications.

Thus, cefepime/enmetazobactam is administered intravenously (0.5 g of enmetazobactam and 2 g of cefepime per vial) as a standard 2 h infusion in patients with cUTIs, including pyelonephritis, and as a standard 4 h infusion in patients with HAP and VAP, regardless of renal function [21]. It is mainly excreted via the kidneys and, thus, the dosage requires adjustment according to the patient’s renal function [21].

The recommended frequency of administration of cefepime/enmetazobactam is every 8 h, but in cases where the patient’s estimated glomerular filtration rate (eGFR) is >60, the dosage should be adjusted by administering 1 g/0.25 g of the antibiotic every 8 h. When the eGFR is 15–29 mL/min/1.73 m^2^, the antibiotic should be administered every 12 h in a reduced dose of 1 g/0.25 g. Moreover, when the eGFR is <15 mL/min/1.73 m^2^ or when the patient is on hemodialysis, cefepime/enmetazobactam is administered once per day in the same dose of 1 g/0.25 g [21]. For patients undergoing continuous renal replacement therapy (CRRT), the recommended therapeutic dose is higher than the dose in patients undergoing hemodialysis. On the other hand, if the eGFR is ≥130 mL/min/1.73 m^2^, a 4 h intravenous infusion is recommended instead of the standard 2 h infusion (Table 2). Renal function should be supervised closely, and the dosage should be adjusted accordingly. Usually, the duration of treatment with cefepime/enmetazobactam is between 7 and 14 days [21].

It is worth mentioning that only one phase 3 clinical trial has been performed to date: the “ALLIUM” trial, which is a randomized, double-blind, multi-center clinical study comparing cefepime/enmetazobactam to piperacillin/tazobactam in patients with a cUTI or pyelonephritis [24]. A total of 1041 patients were enrolled, and 1034 received at least one dose of either cefepime/enmetazobactam or piperacillin/tazobactam. A primary analysis was performed in 678 individuals who received at least one dose of the antibiotics and had a sensitive Gram-negative pathogen to both antibiotics in the urine or both in the urine and blood [more than 10^5^ colony-forming units (CFU)/mL] [24].

The primary outcome was a combined endpoint: complete resolution of signs and symptoms (clinical cure) along with pathogen eradication (less than 10^3^ CFU/mL, microbiological cure) at day 14. The noninferiority of cefepime/enmetazobactam was proven since 79.1% of participants of this group, compared to 58.9% of the piperacillin/tazobactam-treated group, fully recovered in the primary analysis [95% CI (14.3% to 27.9%)], a difference that met the −10% noninferiority pre-established margin by the authors [24]. Cefepime/enmetazobactam also met the criteria for superiority compared to piperacillin/tazobactam, as far as the primary outcome is concerned, since the overall success of the treatment at day 14 differs between the two agents by 21.2% [95% CI (14.3 to 27.9)]; again, the superiority comparison was also prespecified [24].

Secondary outcomes showed that there were no significant differences in the clinical cure rates, regardless of the microbiological eradication, between the two groups (92.5% for cefepime/enmetazobactam vs. 88.9% for piperacillin/tazobactam). However, the group treated with cefepime/enmetazobactam presented with a higher microbiological cure compared to piperacillin/tazobactam (82.9% vs. 64.9%, respectively; treatment difference 19% [95% CI (12.3% to 25.4%)] [24].

The microbiological species found in the study were mostly *Escherichia coli*, with a similar percentage in the two groups, while other pathogens found in the urine cultures included *Klebsiella pneumoniae*, *Proteus mirabilis*, and *Enterobacter cloacae.* The percentage of ESBLs was similar between the two groups (22% in the cefepime/enmetazobactam group vs. 19.8% in the piperacillin/tazobactam group), but the percentage achieving the primary outcome was higher among the cefepime/enmetazobactam group (73.7% vs. 51.5%, respectively). Emergent safety issues presented in 50% of cefepime/enmetazobactam-treated patients compared to 44% of those taking piperacillin/tazobactam [24].

The most common adverse effects are elevated transaminases, headache, diarrhea, and phlebitis at the injection site (approximately 1 to 10 people). Other side effects that may occur include *Clostridium-difficile*-related diarrhea (approximately 0.2%), candidiasis, a positive direct Coombs test with or without hemolysis, prothrombin-time and partial-thromboplastin-time prolongation, eosinophilia, increased amylase, increased lipase, increased lactate dehydrogenase, and rash. Hypersensitivity reactions have also been reported, as they have with so many other drugs [21].

In conclusion, cefepime/enmetazobactam is a novel combination of the fourth-generation cephalosporin cefepime and the new β-lactamase inhibitor enmetazobactam. Cefepime/enmetazobactam mostly targets ESBLs produced by *Pseudomonas aeruginosa* and Enterobacterales [25]. Taking into account that resistance to older β-lactam/β-lactamase inhibitor combination agents is constantly increasing worldwide, this promising combination could be a meropenem-sparing agent in the treatment of patients with cUTIs, including pyelonephritis [19]. Previous studies have already proposed β-lactam/β-lactamase inhibitor combination antibiotics as alternative options to carbapenems for the treatment of patients with infections due to ESBL-producing bacteria (when there is a compatible antibiogram) [26].

The wide use of the antibiotic may be limited by its cost, as with other newly approved antibiotics, and its narrow indication for targeting almost exclusively ESBL-producing Gram-negative pathogens [19]. In Europe, cefepime/enmetazobactam has also been approved as an alternative treatment for difficult-to-treat HAP, VAP, and bacteremia associated with these infections, which offers a useful option to clinicians in countries with a high incidence of infections due to ESBL-producing bacteria, such as Greece [27]. More studies are needed to compare the efficacy of cefepime/enmetazobactam to meropenem in the treatment of patients with the diseases listed above.

## 4. Aztreonam/Avibactam

Aztreonam is the first monocyclic β-lactam (i.e., monobactam) antibiotic to be used in clinical practice. Like other β-lactam antibiotics, it is an inhibitor of bacterial-cell-wall synthesis [28]. Historically, aztreonam has been used for the treatment of patients with aerobic Gram-negative infections of the urinary tract, the abdomen, the skin, the respiratory tract, the endometrium, and bloodstream infections [29]. Nevertheless, during the last few years, the use of this antibiotic has been limited mainly due to the global spread of MDR bacteria and, more specifically, the ESBLs and AmpC-producing bacteria [30].

Although most β-lactam antibiotics are susceptible to MBLs, aztreonam resists hydrolysis by MBLs [31]. This useful characteristic has prompted researchers to explore combinations of aztreonam with new β-lactamase inhibitors to develop an antibiotic combination with a broader antimicrobial spectrum [31]. However, some pathogens may be resistant to aztreonam via the production of other enzymes (β-lactamases) apart from MBLs and/or other mechanisms of antimicrobial resistance [32].

Avibactam is a novel broad-spectrum β-lactamase inhibitor with significant activity against class A, C, and some D β-lactamases [33,34]. It was developed by Actavis in collaboration with AstraZeneca (and approved by the FDA in 2015) for treating cUTIs and complicated intra-abdominal infections (cIAIs) caused by MDR Gram-negative bacteria [35].

Aztreonam/avibactam is a new combination drug developed by Pfizer (New York, NY, US), under the trade name Emblaveo^®^ [36]. The therapeutic indications of this antibiotic are cIAIs (which are infections expanding beyond the perforated viscus and leading to peritonitis or abscess formation), HAP, VAP, and cUTIs including pyelonephritis (Table 1). It is worth mentioning that aztreonam/avibactam is also indicated for any infection caused by aerobic Gram-negative pathogens in adults with limited therapeutic options [36]. It is not effective against Gram-positive bacilli, anaerobic bacteria, or *Acinetobacter* spp.; so, there is usually a need for an add-on antibiotic in cases where more than one pathogen is suspected [37,38].

The drug is administered intravenously, and each vial contains 1.5 g of aztreonam and 0.5 g of avibactam [38]. It is excreted via the kidneys, so renal function should be closely monitored, and the dosage should be adjusted accordingly. When the estimated creatinine clearance (CrCL, using the Cockcroft–Gault formula) is >50 and ≤80 mL/min, no dose adjustment is needed [38]. It is recommended that the infusion time is 3 h and the interval between doses is 6 h [38]. In that case, a loading dose is given at first, which is followed by maintenance doses beginning at the next dose [38].

For patients with a cIAI, the loading dose is 2 g/0.67 g, while the maintenance dose is 1.5 g/0.5 g, and the treatment lasts 5–10 days; metronidazole is suggested as an additional drug to the treatment when anaerobic bacteria are known or suspected [38]. The dosage and duration of the treatment are the same for cUTIs, including pyelonephritis [38]. For patients with HAP and VAP, the dosage is also the same, but the duration of the treatment differs, lasting 7–14 days [38]. When aztreonam/avibactam is administered in patients with aerobic Gram-negative infections with limited therapeutic options, the dose is the same as previously mentioned, and the duration can last up to 14 days in accordance with the site of infection [38].

When the estimated CrCL is ≤50 mL/min, the dose should be adjusted [38]. When the CrCL is >30 mL/min and ≤50 mL/min, the loading dose is 2 g/0.67 g followed by maintenance doses of 0.75 g/0.25 g every 6 h [38]. When the CrCL is >15 and ≤30 mL/min, the loading dose is 1.35 g/0.45 g, followed by maintenance doses of 0.675 g/0.225 g every 8 h [38]. Lastly, when the CrCL is ≤15 mL/min (on intermittent hemodialysis), the loading dose is 1 g/0.33 g, followed by maintenance doses of 0.675 g/0.225 g every 12 h (Table 2) [38].

Aztreonam/avibactam can be used in patients undergoing hemodialysis. Because both aztreonam and avibactam are removed by the dialysis filter, the drug should be administered after the session of hemodialysis [38]. There is no sufficient data for dosing adjustments for other renal replacement therapies in patients with end-stage renal disease [38].

Pfizer conducted two multicenter, open-label, randomized, prospective, central assessor-blinded, phase 3 studies, named “REVISIT” and “ASSEMBLE”, which evaluated the efficacy, safety, and tolerability of aztreonam/avibactam in patients with severe MDR bacterial Gram-negative infections. The “REVISIT” study included a subset of patients with infections due to isolates producing MBLs [39]. This study compared the outcomes of patients with cIAIs, HAP, and VAP who were treated with aztreonam/avibactam (with or without metronidazole) or meropenem (with or without colistin) [39]. In the intention-to-treat (ITT) analysis, in the subset of patients with a cIAI, cure was observed in 76.4% in the aztreonam/avibactam group vs. 74% in the meropenem group. In the clinically evaluable (CE) analysis, cure was observed in 85.1% [95% CI (79.2 to 89.9)] and 79.5% [95% CI (69.9 to 87.1)] of patients in the aztreonam/avibactam and the meropenem groups, respectively [39].

For patients with HAP or VAP, cure in the ITT analysis was 45.9% in the aztreonam/avibactam (with or without metronidazole) group vs. 41.7% in the meropenem (with or without colistin) group [39]. In the CE analysis of this group, cure was observed in 46.7% of patients [95% CI (32.7 to 61.1)] for the aztreonam/avibactam group vs. 54.5% [95% CI (34.3 to 73.7)] for the meropenem group [39].

The 28-day all-cause mortality was 1.9% for aztreonam/avibactam (with or without metronidazole) compared to 2.9% for meropenem (with or without colistin) for patients with a cIAI; for patients with HAP and VAP, mortality was 10.8% vs. 19.4%, respectively [39]. In general, the novel drug combination was well tolerated, presenting similar adverse effects to aztreonam monotherapy [39]. The rates of severe adverse reactions between the two groups were comparable: 19.3% with aztreonam/avibactam vs. 18.2% with meropenem [39].

The second study, named “ASSEMBLE”, showed that 41.7% of the patients (5/12) receiving aztreonam/avibactam with or without metronidazole due to confirmed MBL-producing Gram-negative bacteria were cured vs. 0% (0/3) of those treated with the best available treatment [40]. Safety issues were found in line with the “REVISIT” study [41]. It is worth mentioning that the manufacturers mentioned that those studies were not designed to test efficacy or to make conclusions; however, they do show the first signs of efficacy and safety of aztreonam/avibactam [41].

The “REJUVENATE” phase 2a study suggested a loading dose of 500/167 mg over a 30 min infusion followed by maintenance doses of 1500/500 mg every 6 h (over 3 h infusions) in patients with normal renal function. This open-label, multicenter study enrolled 34 individuals with a cIAI, grouped into three cohorts, and examined different loading doses followed by different maintenance doses. Patients of cohort 1 received a loading dose of aztreonam/avibactam of 500/137 mg (over 30 min) followed by a maintenance dose of 1500/410 mg administered in a 3 h infusion every 6 h. Patients of cohort 2 received a loading dose of the antibiotic of 500/167 mg (over 30 min) followed by a maintenance dose of 1500/500 mg administered in a 3 h infusion every 6 h. Patients of cohort 3 received aztreonam/avibactam at a higher loading dose for a longer time (1500/500 mg over 3 h) followed by a maintenance dose of 750/250 mg administered in a 3 h infusion every 6 h [42].

Aztreonam/avibactam was approved in Europe in April 2024 for the treatment of patients with cIAIs, HAP, VAP, cUTIs including pyelonephritis, and for infections caused by aerobic Gram-negative bacteria in patients with limited treatment options [43]. However, in the United States, this novel combination agent has not been approved yet, since the results of phase 3 clinical trials have not been published to date (Table 1) [44]. Nevertheless, in the U.S., it is accepted to use aztreonam separately along with the combination drug ceftazidime/avibactam when indicated [44].

The most common side effects are dizziness, nausea, diarrhea (in cases of *Clostridium difficile,* immediate discontinuation of the treatment is recommended), abdominal pain, vomiting, rash, elevated liver enzymes, anemia, thrombocytosis, thrombocytopenia, confusion, phlebitis at the injection site, thrombophlebitis, injection-site pain, extravasation, and pyrexia [38].

In conclusion, aztreonam/avibactam is a novel drug targeting Gram-negative bacteria, which is used in patients with cIAIs, HAP, VAP, and cUTIs including pyelonephritis [43]. This novel combination agent is active against carbapenem-resistant Enterobacterales, including those that produce ESBLs, MBLs, and serine carbapenemases (Table 1) [39]. Lastly, it can also be used to treat patients infected with aerobic Gram-negative bacteria who have limited treatment options [36].

## 5. Sulbactam/Durlobactam

Sulbactam is a β-lactam-ring-containing β-lactamase inhibitor [45]. The β-lactam ring gives sulbactam an intrinsic weak antibacterial activity in contrast to other β-lactamase inhibitors, which need to bind to the β-lactam to exhibit their antibacterial capacities [45].

Durlobactam is a non-β-lactam, β-lactamase inhibitor, which, when combined with sulbactam, protects the latter from degradation by specific serine β-lactamases (mostly by OXAs) produced by the *Acinetobacter baumannii–calcoaceticus* complex (*Acinetobacter baumannii*, *Acinetobacter nosocomialis*, *Acinetobacter pitti*, *Acinetobacter seifertii*) [33,46,47]. Durlobactam is a chemical derivative of avibactam [48].

Sulbactam/durlobactam received approval in May 2023 from the FDA for use in adult patients (>18 years old) with hospital-acquired bacterial pneumonia (HABP) and ventilator-associated bacterial pneumonia (VABP) due to isolates of the *Acinetobacter baumannii–calcoaceticus* complex [49]. Entasis Therapeutics Inc. (Waltham, MA, US) developed the antimicrobial combination, and it is sold under the brand name Xacduro^®^ [49]. However, at the time of writing, the combination has not yet received approval in Europe (Table 1).

Sulbactam/durlobactam is an interesting combination as it consists of two β-lactamase inhibitors (Table 1). It can be used for patients with infections due to isolates with advanced resistance patterns, namely carbapenem-resistant *Acinetobacter baumannii* (CRAB), given that its efficacy in treating patients with severe pneumonia caused by CRAB has been shown in the ATTACK study [45,50].

Sulbactam/durlobactam is excreted via the kidneys, so renal function should be monitored closely, and the frequency of dosing should be adjusted accordingly. The agent exists only in an intravenous formulation [51]. Thus, it is recommended that the dose of the agent is 1/1 (sulbactam/durlobactam) g, which is administered over 3 h to all patients, but the frequency differs according to the patient’s renal function.

Sulbactam/durlobactam should be administered every 6 h in patients with a CrCL of 45–129 mL/min (standard administration), every 8 h in patients with a CrCL of 30–44 mL/min, every 4 h in patients with a CrCL of ≥130 mL/min, every 12 h in patients with a CrCL of 15–29 mL/min, and once per day if the CrCL is <15 mL/min [except for the first three doses, which should be given every 12 h for patients starting the antibiotic regimen (for patients already receiving the agent but who exhibit a decline in their renal function, the medication should be administered every 24 h)] [51]. It should also be administered after hemodialysis in patients undergoing intermittent hemodialysis (Table 2) [51]. The duration of treatment is 7–14 days, according to the patient’s clinical status.

A multicenter, open-label, active-controlled, noninferiority clinical trial, which enrolled 177 hospitalized adults who were hospitalized due to pneumonia caused by carbapenem-resistant *Acinetobacter baumannii*, examined the efficacy of sulbactam/durlobactam [45]. The subjects received either sulbactam/durlobactam or colistin for up to 14 days. All patients also received imipenem/cilastatin as supplementary therapy for potentially different causative pathogens other than *Acinetobacter baumannii* [45].

The primary endpoint was 28-day all-cause mortality in patients with confirmed *Acinetobacter baumannii* infection; 12/63 (19%) of those receiving sulbactam/durlobactam died vs. 20/62 (32%) of those receiving colistin, suggesting that the former was noninferior to colistin regarding efficacy (pre-established noninferiority margin of 20% by the authors) [45].

Sulbactam/durlobactam also showed a significant advantage in nephrotoxicity (measured by the RIFLE criteria) compared to colistin; 13% of patients treated with sulbactam/durlobactam presented with nephrotoxicity compared to 38% of those treated with colistin (approximately a 24% difference, *p <* 0.001) [45].

Common adverse effects of sulbactam/durlobactam include anemia, hypokalemia, elevated transaminases, diarrhea (including cases related to *Clostridium difficile*), and hypersensitivity. Thus, patients presenting with a history of severe hypersensitivity to β-lactams should not use this agent [51]. Further studies of sulbactam/durlobactam are needed in larger populations designed with narrower noninferiority margins, along with the clinical data from the physicians who used this novel antibiotic in their patients.

## 6. Conclusions

MDR bacteria have become a global health concern, causing millions of deaths annually. Recently approved β-lactam/β-lactamase inhibitor combination antibiotics, such as cefepime/enmetazobactam (targeting ESBLs produced by *Pseudomonas aeruginosa* and Enterobacterales), aztreonam/avibactam (targeting carbapenem-resistant Enterobacterales, including those that produce ESBLs, MBLs, and serine carbapenemases), and sulbactam/durlobactam (targeting the *Acinetobacter baumannii–calcoaceticus* complex) demonstrate considerable therapeutic advances.

However, the new β-lactam/β-lactamase inhibitor combination antibiotics have not become widely used for several reasons, including the limited relevant clinical data and, at least in some settings, their considerable cost. In particular, the limited trial data may have contributed to different approval statuses and indications for these agents between Europe and the United States. Also, the unavailability of potentially useful new agents may complicate the control of infections due to MDR pathogens. Additional studies with larger patient numbers (and subsequently with narrower noninferiority margins for some agents) and published post-clinical-trial results are needed to evaluate the effectiveness of these recently introduced combination antibiotics to guide their optimal use in combating MDR pathogens. Lastly, efforts to investigate new therapeutic antibiotic combinations, such as sulbactam/relebactam and sulbactam/avibactam, especially against CRAB infections, are underway, as well as several β-lactam/β-lactamase inhibitor combination antibiotics that are in clinical phases of development. [48,52].

## Figures and Tables

**Table 1:** New β-lactam/β-lactamase inhibitor combination antibiotics ^a^.

Generic Name	Brand Name	Drug Approval	Antibiotic Class	Antimicrobial Spectrum	Site of Infection
Cefepime/enmetazobactam	EXBLIFEP^®^	FDA, EMA	fourth-generation cephalosporin/penicillin acid sulfone	ESBL producing *Pseudomonas aeruginosa* and Enterobacterales (*Escherichia coli*, *Klebsiella pneumoniae*, *Proteus mirabilis*, and *Enterobacter cloacae*)	cUTI, including pyelonephritis (FDA, EMA) HAP, VAP (and bacteremia associated with those infections) (EMA) ^b^
Aztreonam/avibactam	EMBLAVEO^®^	EMA	monocyclic β-lactam /broad-spectrum β-lactamase inhibitor	carbapenem-resistant Enterobacterales (including those that produce ESBLs, serine carbapenemases, and metallo-β-lactamases)	cIAI, cUTI including pyelonephritis, HAP, VAP, and aerobic Gram-negative infections ^b^
Sulbactam/durlobactam	XACDURO^®^	FDA	β-lactamase inhibitor/β-lactamase inhibitor	*Acinetobacter baumannii-calcoaceticus* complex	HABP and VABP in patients older than 18 years

^a^ Abbreviations: cIAI, complicated intra-abdominal infection; cUTI, complicated urinary tract infection; HABP, hospital-acquired bacterial pneumonia; VABP, ventilator-associated bacterial pneumonia; HAP, hospital-acquired pneumonia; VAP, ventilator-associated pneumonia; MDR, multidrug-resistant; FDA, Food and Drug Administration; EMA, European Medicines Agency. ^b^ in adults with limited therapeutic options.

**Table 2:** Recommended dose adjustments of new β-lactam/β-lactamase inhibitor combination antibiotics based on the degree of renal impairment ^a^.

eGFR (mL/min/1.73 m^2^) or Calculated CrCI (mL/min)	Cefepime/ Enmetazobactam (2 g/0.5 g) ^b^	Aztreonam/ Avibactam	Sulbactam/ Durlobactam (1 g/1 g)
>130	q8h *	NA	q4h
50–130	2 g/0.5 g, q8h (when eGFR 60–130 mL/min/1.73 m^2)^	2 g/0.67 g, as a loading dose, then 1.5 g/0.5 g q6h	q6h
30–50	1 g/ 0.25 g q8h (when eGFR 30–60 mL/min/1.73 m^2^)	2 g/0.67 g, as a loading dose, then 0.75 g/0.25 g q6h	q8h
15–30	1 g/0.25 g q12h	1.35 g/0.45 g, as a loading dose, then 0.675 g/0.225 g q8h	q12h
<15	1 g/0.25 g q24h	1 g/0.33 g as a loading dose, then 0.675 g/0.225 g q12h	q24h

^a^ Abbreviations: eGFR, estimated glomerular filtration rate; CrCl, creatinine clearance; q4h, every 4 h; q8h, every 8 h, q12h, every 12 h; q24h, every 24 h; NA; non available; ^b^ 2-hour infusion in cUTIs, and 4-h infusion in patients with HAP and VAP; * 4-h infusion.

## Data Availability

Not applicable.

## Abbreviations

AmpC	Ampicillinase C
BL/BLI	β-lactam/β-lactamase inhibitor
CE	Clinically evaluable
CFU	Colony-forming units
CRAB	carbapenem-resistant *Acinetobacter baumannii*
CrCL	Creatinine clearance
CRRT	Continuous renal replacement therapy
cUTI	Complicated urinary tract infection
cIAI	Complicated intra-abdominal infection
eGFR	Estimated glomerular filtration rate
ESBL	Extended-spectrum β-lactamases
FDA	Food and Drug Administration
HAP	Hospital-acquired pneumonia
HABP	Hospital-acquired bacterial pneumonia
ITT	Intention to treat
KPC	Klebsiella pneumoniae carbapenemase
MBL	Metallo-β-lactamase
MDR	Multidrug resistance
MIC	Minimum inhibitory concentration
OXA	Oxacillinase
PBP	Penicillin-binding proteins
VAP	Ventilator-associated pneumonia
VABP	Ventilator-associated bacterial pneumonia
